# Automatic Point Cloud Patching of Intraoral Three-Dimensional Scanning Based on Deep Learning

**DOI:** 10.1016/j.identj.2025.100911

**Published:** 2025-07-19

**Authors:** Qianhan Zheng, Yimin Wang, Mengqi Zhou, Yongjia Wu, Jiahao Chen, Xiaozhe Wang, Lixia Gao, Ting Kang, Xuepeng Chen, Weifang Zhang

**Affiliations:** a Stomatology Hospital, School of Stomatology, Zhejiang University School of Medicine, Clinical Research Center for Oral Diseases of Zhejiang Province, Key Laboratory of Oral Biomedical Research of Zhejiang Province, Cancer Center of Zhejiang University, Hangzhou, Zhejiang, China; b School of Stomatology, Zhejiang Chinese Medical University, Hangzhou, Zhejiang, China; c Savaid Stomatology School, Hangzhou Medical College, Hangzhou, Zhejiang, China; d Social Medicine & Health Affairs Administration, Zhejiang University, Hangzhou, Zhejiang, China

**Keywords:** Intraoral scan, Point cloud, Deep learning, Completion

## Abstract

**Introduction and aims:**

Intraoral scanning (IOS) captures real-time surface morphology of teeth and is widely used in clinical dentistry. However, due to the complex intraoral environment, data loss during scanning is common, leading to incomplete three-dimensional (3D) point clouds. This study aimed to develop and evaluate a deep learning–based method to automatically restore missing regions in intraoral 3D point clouds, thereby improving the accuracy and efficiency of digital orthodontic workflows.

**Methods:**

A Point Fractal Network architecture was adopted to reconstruct incomplete IOS data. A dataset comprising 314 IOS scans and 4162 individual teeth was used for training and validation. Missing data were simulated by removing random portions of point clouds (5%, 10%, 15%, and 20%). Model performance was assessed using Chamfer distance (CD) to measure the accuracy of point cloud completion across different levels of data loss.

**Results:**

The proposed method achieves robust performance, maintaining average CD values below 0.01 across most levels of simulated data loss. Visual comparisons confirmed high geometric fidelity between the completed and original point clouds. Furthermore, the model demonstrated efficient processing, completing each point cloud in approximately 0.5 seconds, enabling near real-time restoration during clinical scanning.

**Conclusion:**

The deep learning–based model accurately restores missing IOS data, improving the precision and efficiency of digital dental workflows. Its speed and accuracy support real-time clinical applications and reduce reliance on manual corrections.

**Clinical Relevance:**

This method improves clinical efficiency, reduces chairside time, and enhances both patient comfort and treatment acceptance. In addition, it minimises human error and increases the precision of dental restorations. As digital dentistry continues to evolve, this approach holds great potential for improving the accuracy and efficiency of dental treatments, paving the way for broader artificial intelligence integration in clinical practice.

## Introduction

Dental impressions are a routine procedure in clinical dentistry used to record and transfer the shape of the oral soft and hard tissues for subsequent diagnosis, treatment planning, and fabrication of dental prosthetics.^1^ For decades, traditional gypsum models have been the standard practice.^2^ However, certain steps involved in their preparation – such as mixing impression materials, disinfection, storage, transportation, and the production of gypsum casts – can introduce cumulative inaccuracies, potentially affecting the precision of the final dental model.^3^

In recent years, many commercial intraoral scanners have been introduced. These devices utilise optical scanning technology to project light onto the surface of the teeth and capture the reflected light, generating digital dental impressions in real time.^4^ Research has shown that replacing traditional impressions with intraoral scanning (IOS) can reduce patient discomfort,^5^ improve environmental sustainability, and lower the risk of model damage or deformation.^6^ In addition, digital models generated by these scanners can be visualised and magnified in real time, with features like automatic colour scanning for aesthetic shade matching.^7^ These advantages facilitate treatment planning, enhance patient acceptance, improve communication with dental laboratories, shorten procedure times, and minimise storage requirements.^8^

When acquiring and reconstructing digital dental impressions, the data obtained from IOS are typically represented in the form of three-dimensional (3D) point clouds that capture the shape of the dental arch. A point cloud is a set of data points in space, each characterised by its basic 3D coordinates and additional information such as reflectivity, reflection intensity, distance to the scanner centre, horizontal and vertical angles, and deviation values.^9^ As a common representation of 3D data, point clouds retain the original geometric information in 3D space without discretisation,^10^ allowing for an intuitive and accurate simulation of the complex surface structures of both the dental hard tissues (such as crowns) and soft tissues (such as gingiva).

However, due to the confined space and complex environment in the oral cavity (such as moving mucosa, saliva, oral humidity, and tongue movement), as well as hand tremors when using the scanner, the point cloud data of the scanned areas often suffer from issues like overexposure or shadow effects.^11^ This results in incomplete point clouds that lack sufficient features, leading to gaps in the reconstructed 3D model.^12^ Such issues can affect the accuracy of the final digital dental model, requiring the clinician to repeatedly scan the same area to obtain complete point cloud data, thus lowering the precision and efficiency of clinical work. Therefore, dynamically repairing incomplete 3D point cloud data obtained from IOS is a critical task. Successful automation of this repair process would greatly improve the accuracy of 3D scans, reduce the frequency of repeated scans, and streamline the entire scanning workflow.^13^

Currently, with the rapid development of artificial intelligence,^14^ many studies have applied deep learning techniques to the intelligent processing of 3D point cloud data, such as feature extraction and image processing.^15^ Since 3D point clouds are unstructured and unordered, most previous deep learning–based methods converted point clouds into image sets (such as images from different viewpoints) or regularised voxel representations.^16^ However, such multiview or voxel-based representations can lead to excessive data sizes and limit the output resolution.^17^

With the advancement of deep learning for point clouds, the introduction of PointNet in 2017,^18^ and its subsequent improvement PointNet++,^19^ has provided a ground-breaking method that directly computes from raw point clouds without requiring data conversion. These methods leverage three key properties unique to point cloud data: invariance to affine and rigid transformations, the unordered nature of the points, and the spatial relationships between points. Since then, various point cloud learning methods based on PointNet and PointNet++ as encoders have emerged.^20^, ^21^, ^22^ By incorporating modules such as hierarchical convolutions or edge convolutions into the PointNet structure, these methods attempt to address the ordering problem of point clouds and preserve local geometric structures, thereby significantly improving the accuracy and robustness of point cloud feature extraction and recognition.

In the field of 3D IOS data processing, deep point cloud learning has been widely applied to tasks such as crown recognition and segmentation,^23^ as well as the intelligent generation of dental restorations,^24^ demonstrating its strong generalisation capabilities. Building on these applications, this study proposes and validates a fully automated deep learning–based method for completing 3D IOS point cloud data. The proposed method is primarily based on the Point Fractal Network (PF-Net) architecture,^25^ which captures the feature information of real intraoral dental point clouds to predict and fill in missing regions, generating a refined and complete model.

## Methodology

The study was retrospective in design and was reviewed and approved by the Ethics Committee of the Affiliated Hospital of Stomatology, School of Stomatology, Zhejiang University School of Medicine (2024-001[R]). All images were essential for model training and clinical validation, ensuring that patient-specific information was anonymised in compliance with ethical standards.

The dataset included 314 IOS images (157 upper jaws and 157 lower jaws) acquired by an iTero Element intraoral scanner (Align Technologies) between January 2023 and November 2024 from the Affiliated Hospital of Stomatology, School of Stomatology, Zhejiang University School of Medicine. All collected data underwent manual review and screening, and only scans with complete coverage, clear resolution, and no significant noise or occlusion were included. The followi ng types of data were excluded: scans with extensive occlusions or missing regions; cases with unstable point cloud reconstructions caused by saliva, soft tissue movement, or operational artefacts; and scans with severe malocclusion or crowding that prevented clear identification of individual tooth crowns.

The obtained IOS data were first processed using the MeshSegNet model developed by Lian et al^26^ for the automatic recognition and segmentation of individual tooth crowns. The automatically segmented results were subsequently reviewed and manually refined by a licensed dentist to ensure anatomical accuracy and consistent labelling.

The training dataset included teeth from the upper and lower central incisors to the second molars. Only teeth with complete scans and clear morphology were retained to ensure high-quality ground truth labels. Teeth exhibiting artefacts, severe wear, abnormal morphology, or extensive loss due to caries or impaction were excluded from the dataset.

After preliminary sample collection and filtering, a total of 4162 teeth were included in the dataset, which were categorised into 10 classes based on tooth position: upper central incisors (310), upper lateral incisors (301), upper canines (300), upper premolars (611), upper molars (536), lower central incisors (289), lower lateral incisors (310), lower canines (313), lower premolars (609), and lower molars (583). The teeth in each class were randomly divided into three subsets: the training set, used to train the point cloud completion models; the validation set, used to assess model performance and optimise hyperparameters; and the test set, used to evaluate the model's performance compared to the ground truth. The proportions for the training, validation, and test sets were 7:2:1.

**Fig. 1:**
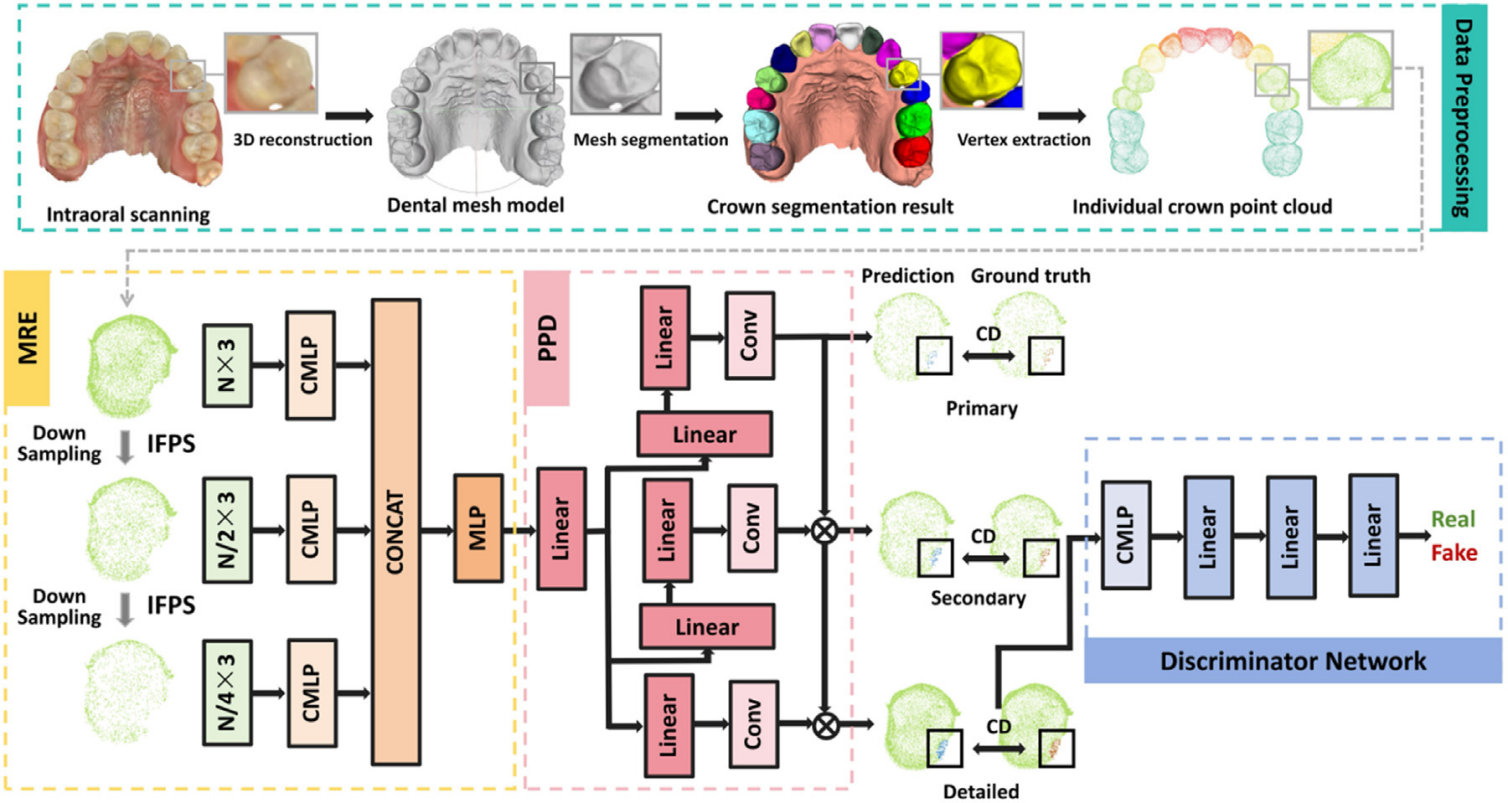
A schematic diagram of our proposed method. CD, Chamfer distance; CMLP, Conditional Multi-Layer Perceptron; CONCAT, Concatenation; Conv, Convolution; IFPS, Iterative Farthest Point Sampling; MRE, multi-resolution encoder; PPD, point pyramid decoder.

A schematic diagram outlining our methodology is depicted in Figure 1. Unlike other point cloud completion algorithms,^27^, ^28^, ^29^ which often alter the overall shape of the point cloud and introduce noise or geometric distortions, the proposed method preserves the spatial arrangement of the incomplete point cloud while accurately reconstructing the detailed geometric structure of the missing regions. It consists of three key components: a multiresolution encoder (MRE) for extracting multiscale features,^30^ a Point Pyramid Decoder (PPD) for hierarchical point generation,^31^ and a Discriminator Network.^32^ The MRE utilises Iterative Farthest Point Sampling^19^ to efficiently select feature points, preserving the shape's core geometry. The PPD improves reconstruction quality by combining low- and high-resolution features to minimise distortions and retain detailed local geometry. In addition, the Discriminator Network, built on the PointNet architecture, learns to distinguish between real point clouds and completed point clouds.

During the model training, multiple loss functions are employed to optimise point cloud completion quality. Discriminator loss (Loss_D) evaluates the discriminator's ability to distinguish between real and generated point clouds, with a lower value indicating a stronger discriminator. Total generator loss (Loss_G) assesses the overall performance of the generator and enhances the realism of generated point clouds by combining Generator adversarial loss (Loss_G_D) and Generator L2 loss (L2_Loss). Chamfer distance loss (CD_Loss)^33^ quantifies the geometric discrepancy between generated and real point clouds, preserving both global structure and fine details.

To train our model, we automatically generate missing parts of the tooth point clouds by randomly selecting one viewpoint as the centre and removing points within a specified radius from the complete crown data. The number of missing points is controlled by adjusting the radius, and we train and test the model on different levels of missing data, using identical training parameters for each scenario. Our network is built using PyTorch, with all 3 modules trained alternately using the adaptive moment estimation optimiser, an initial learning rate of 0.0001, and a batch size of 48. The training was conducted on a CPU (12th Gen Intel Core i5-12600) and a GPU (NVIDIA GeForce RTX 4060). After training for 100 epochs, we select the best-performing model for subsequent accuracy evaluation.

To evaluate the performance of our automated method across different ranges of missing data, we use the Chamfer distance (CD)^34^^,^^35^ and Hausdorff distance (HD)^36^ as evaluation metrics. These two metrics jointly assess the overall deviation and the worst-case deviation between the crown point cloud completion result generated by our model and the ground truth.

For CD, we first compute two key metrics: prediction to ground truth (Pred→GT) error and ground truth to prediction (GT→Pred) error. The Pred→GT error calculates the average squared distance from each point in the predicted point cloud to its nearest point in the ground truth, quantifying the difference between the prediction and the ground truth. The GT→Pred error computes the average squared distance from each point in the ground truth point cloud to its nearest point in the predicted point cloud, measuring how well the predicted point cloud covers the surface of the ground truth. Based on the above statistics, we calculate the overall CD value for each sample, as shown in the formula below:

$$\begin{array}{c} d_{CD}\left(S_{1},S_{2}\right)=d_{CD}\left(S_{1}\to S_{2}\right)+d_{CD}\left(S_{2}\to S_{1}\right)\\ d_{CD}\left(S_{1},S_{2}\right)=\frac{1}{S_{1}}\sum_{x\in S_{1}}\min_{y\in S_{2}}{∥x-y∥}_{2}^{2}+\frac{1}{S_{2}}\sum_{y\in S_{2}}\min_{x\in S_{1}}{∥y-x∥}_{2}^{2} \end{array}$$

Finally, we compute the average CD value for each tooth position at different levels of missing data, which serves as the evaluation criterion for the completion results.

In addition to CD, we incorporate HD to further capture the maximum point-wise deviation between the generated point cloud and the ground truth. Specifically, HD measures the largest distance from a point in one point cloud to its nearest neighbour in the other point cloud. This metric is particularly useful for identifying extreme discrepancies that may not be reflected by average-based metrics like CD. The HD for a sample is computed as:

$$\begin{array}{c} d_{HD}\left(S_{1},S_{2}\right)=max\;\left(d_{HD}\left(S_{1}\to S_{2}\right)\;,\;d_{HD}\left(S_{2}\to S_{1}\right)\;\right)\\ d_{HD}\left(S_{1},S_{2}\right)=max\left(\underset{x\;\in S_{1}}{\text{max}\;}\underset{y\in S_{2}}{\text{min}}∥x-y∥\;,\;\underset{y\;\in S_{2}}{\text{max}\;}\underset{x\in S_{1}}{\text{min}}∥y-x∥\right) \end{array}$$

## Results

**Fig. 2:**
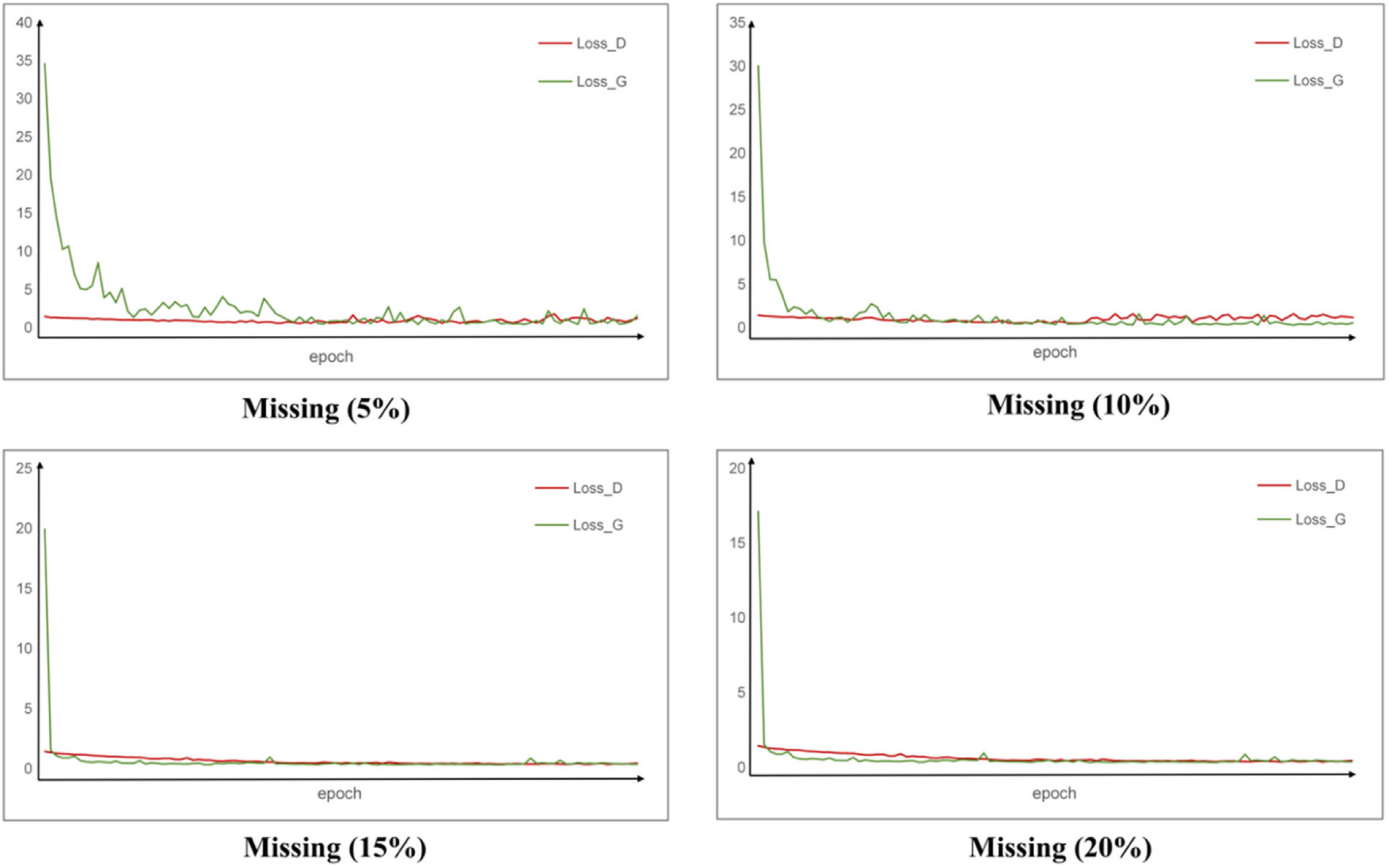
Statistical chart of the generator and discriminator loss during model training. Loss_D, loss of discriminator; Loss_G, loss of generator.

**Fig. 3:**
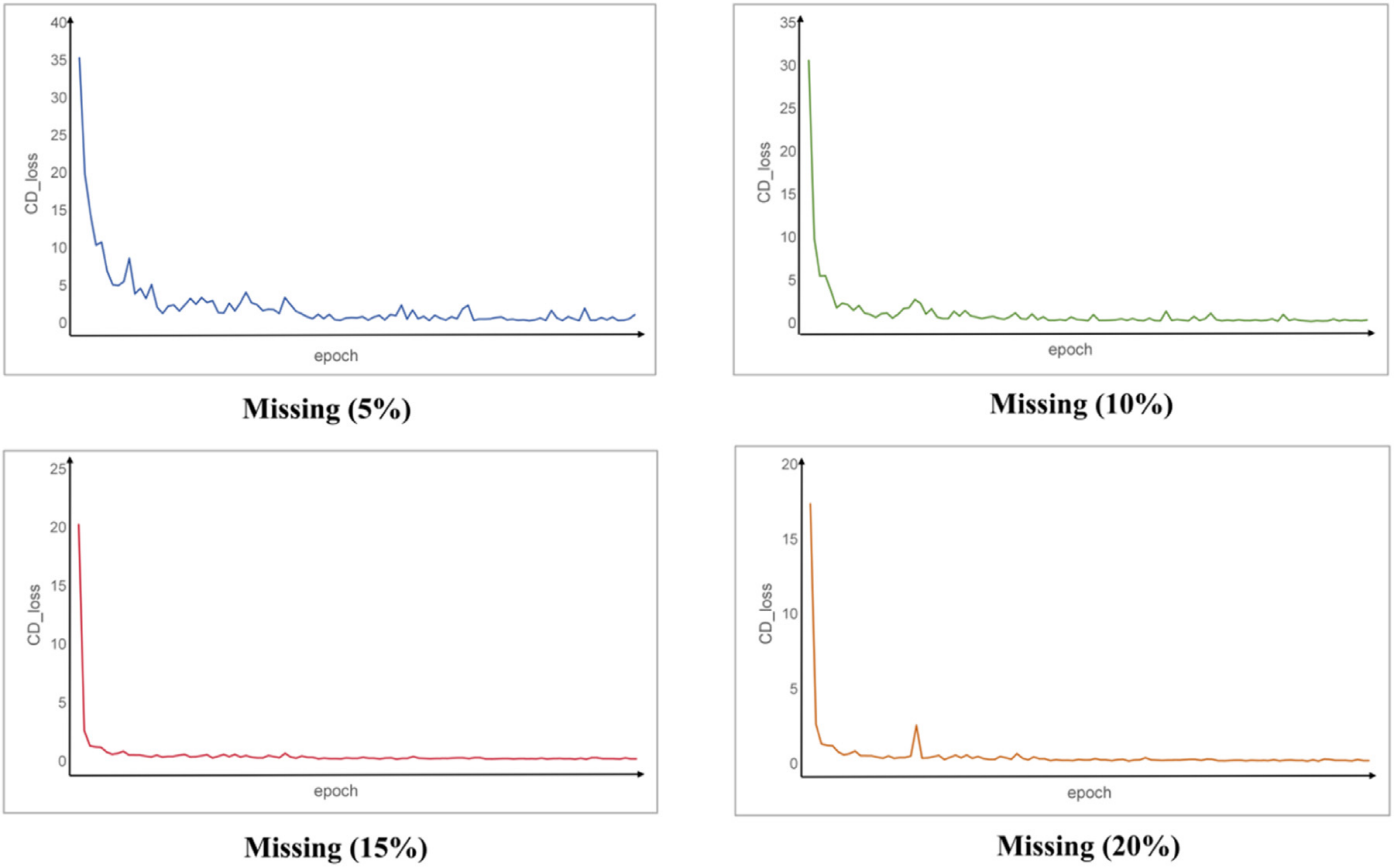
Statistical chart of the CD loss during model training. CD_loss, Chamfer distance loss.

Figures 2 and 3 illustrate the loss variations during model training under different missing rates (5%, 10%, 15%, and 20%). As shown in the figures, the initial loss values across all missing rates are relatively high, indicating that the quality of the generated dental point clouds is poor at the beginning, with significant discrepancies compared to the ground truth. However, as training progresses, both Loss_G and CD_Loss decrease rapidly, suggesting that the model is able to converge quickly in the early stages of training. In the mid-to-late phases, all curves stabilise, indicating that adversarial training has reached a balanced state, allowing the model to effectively learn and complete the missing dental point clouds.

In addition, the figures reveal that as the missing rate increases, the initial values of Loss_G and CD_Loss tend to be lower, with a faster decline and earlier stabilisation. This suggests that the model may more quickly discover a stable completion strategy when dealing with higher missing rates. Moreover, the fluctuation of the loss curves is relatively smaller at 15% and 20% missing rates, whereas it is most pronounced at 5%. This could be due to the fact that at lower missing rates, the generator is more likely to encounter local optima during training, leading to greater loss variations and reduced stability in the completion process.

**Fig. 4:**
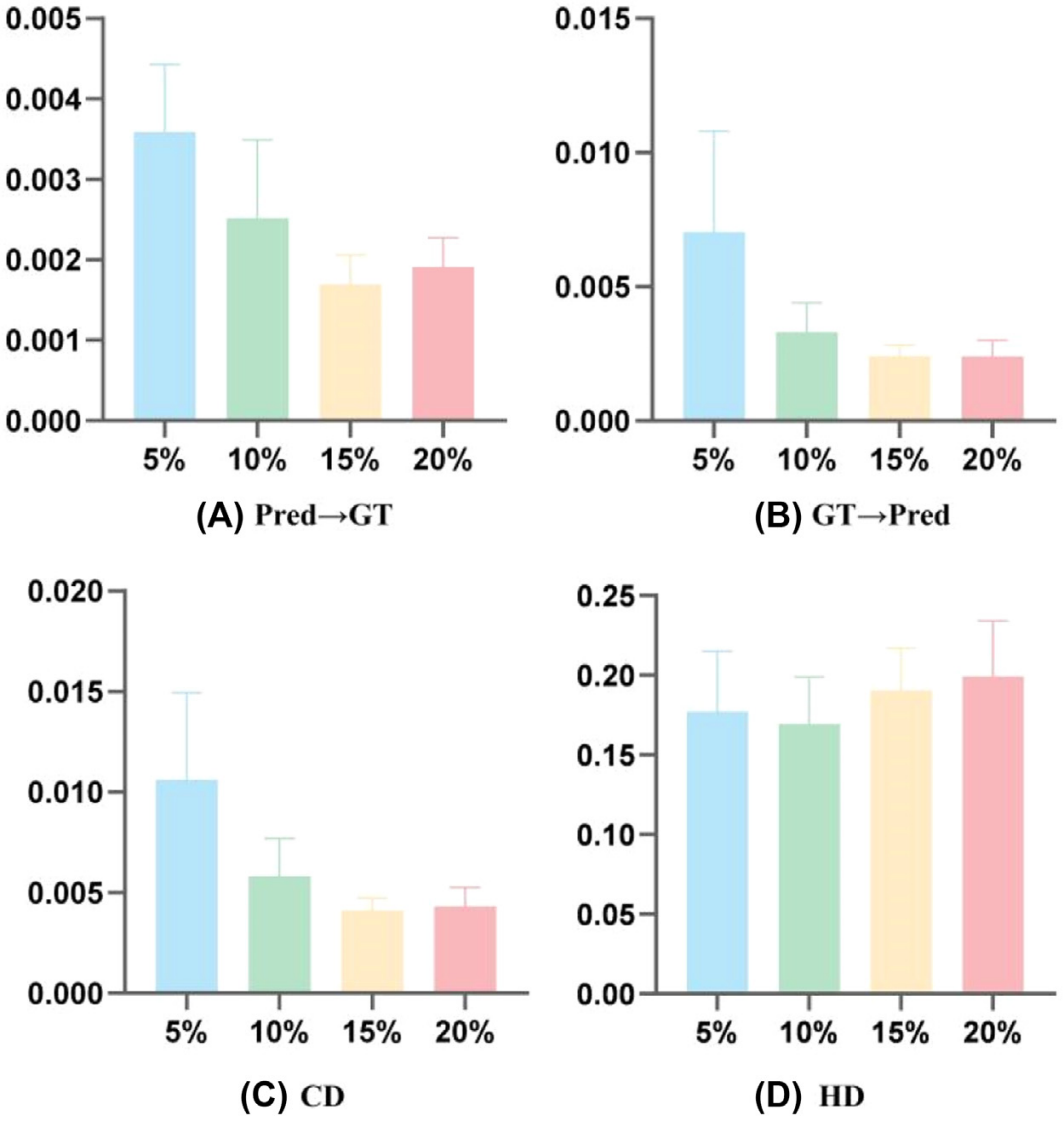
Statistical chart of the performances of the proposed method. CD, Chamfer distance; GT, ground truth; HD, Hausdorff distance; Pred, prediction.

Tables 1 and 2 and Figure 4 are the test results of the PF-Net–based model for crown point cloud completion under different missing proportions (5%, 10%, 15%, and 20%) at various tooth positions. The results demonstrate that except for the 5% missing data proportion, where the average CD value is 0.011 ± 0.0043, the average CD values for all other missing proportions remain below 0.01. Notably, the model achieves the lowest average CD value of 0.0041 ± 0.0006 at the 15% missing data level. In addition, the average HD values across all missing data proportions are below 0.2, with the model attaining the lowest average HD value of 0.1692 ± 0.0296 at the 10% missing data proportion.

**Table 1:** Evaluation results of the proposed method under different missing proportions in terms of Pred→GT and GT→Pred metrics.

Tooth position	Missing (5%)		Missing (10%)	
	Pred→GT	GT→Pred	Pred→GT	GT→Pred
Maxillary central incisor	0.0066	0.0037	0.0027	0.0019
Maxillary lateral incisor	0.0056	0.0032	0.0035	0.0021
Maxillary canine	0.0042	0.0035	0.0022	0.0017
Maxillary premolar	0.0120	0.0032	0.0055	0.0044
Maxillary molar	0.0089	0.0050	0.0031	0.0034
Mandibular central incisor	0.0141	0.0048	0.0037	0.0037
Mandibular lateral incisor	0.0045	0.0035	0.0044	0.0020
Mandibular canine	0.0035	0.0029	0.0019	0.0015
Mandibular premolar	0.0028	0.0022	0.0024	0.0024
Mandibular molar	0.0082	0.0039	0.0037	0.0021
Average	0.0070 ± 0.0037	0.0036 ± 0.0008	0.0033 ± 0.0011	0.0025 ± 0.0010


Tooth position	Missing (15%)		Missing (20%)	
	Pred→GT	GT→Pred	Pred→GT	GT→Pred
Maxillary central incisor	0.0023	0.0017	0.0020	0.0017
Maxillary lateral incisor	0.0027	0.0018	0.0022	0.0018
Maxillary canine	0.0024	0.0017	0.0023	0.0019
Maxillary premolar	0.0029	0.0024	0.0028	0.0021
Maxillary molar	0.0024	0.0021	0.0036	0.0023
Mandibular central incisor	0.0018	0.0012	0.0019	0.0016
Mandibular lateral incisor	0.0031	0.0013	0.0029	0.0026
Mandibular canine	0.0019	0.0016	0.0019	0.0016
Mandibular premolar	0.0025	0.0014	0.0017	0.0014
Mandibular molar	0.0022	0.0018	0.0028	0.0021
Average	0.0024 ± 0.0004	0.0017 ± 0.0004	0.0024 ± 0.0006	0.0019 ± 0.0004

GT, ground truth; Pred, prediction.

**Table 2:** Evaluation results of the proposed method under different missing proportions in terms of CD and HD metrics.

Tooth position	Missing (5%)		Missing (10%)	
	CD	HD	CD	HD
Maxillary central incisor	0.0102	0.1487	0.0046	0.1696
Maxillary lateral incisor	0.0088	0.1578	0.0055	0.1654
Maxillary canine	0.0077	0.1764	0.0039	0.1357
Maxillary premolar	0.015	0.1848	0.0099	0.1727
Maxillary molar	0.0139	0.1983	0.0065	0.1965
Mandibular central incisor	0.0189	0.2707	0.0074	0.2213
Mandibular lateral incisor	0.0080	0.1729	0.0064	0.1941
Mandibular canine	0.0064	0.1433	0.0034	0.1380
Mandibular premolar	0.0050	0.1438	0.0048	0.1283
Mandibular molar	0.0122	0.1761	0.0058	0.1702
Average	0.0106 ± 0.0043	0.1772 ± 0.0376	0.0058 ± 0.0019	0.1692 ± 0.0296


Tooth position	Missing (15%)		Missing (20%)	
	CD	HD	CD	HD
Maxillary central incisor	0.0040	0.2106	0.0037	0.2147
Maxillary lateral incisor	0.0045	0.2020	0.0040	0.2263
Maxillary canine	0.0041	0.2023	0.0042	0.2192
Maxillary premolar	0.0053	0.2159	0.0049	0.2391
Maxillary molar	0.0045	0.2158	0.0059	0.2473
Mandibular central incisor	0.0030	0.1990	0.0035	0.1921
Mandibular lateral incisor	0.0044	0.1964	0.0055	0.1698
Mandibular canine	0.0035	0.1625	0.0035	0.1607
Mandibular premolar	0.0039	0.1394	0.0031	0.1479
Mandibular molar	0.0040	0.1603	0.0049	0.1749
Average	0.0041 ± 0.0006	0.1904 ± 0.0266	0.0043 ± 0.0009	0.1992 ± 0.0348

CD, Chamfer distance; HD, Hausdorff distance.

**Fig. 5:**
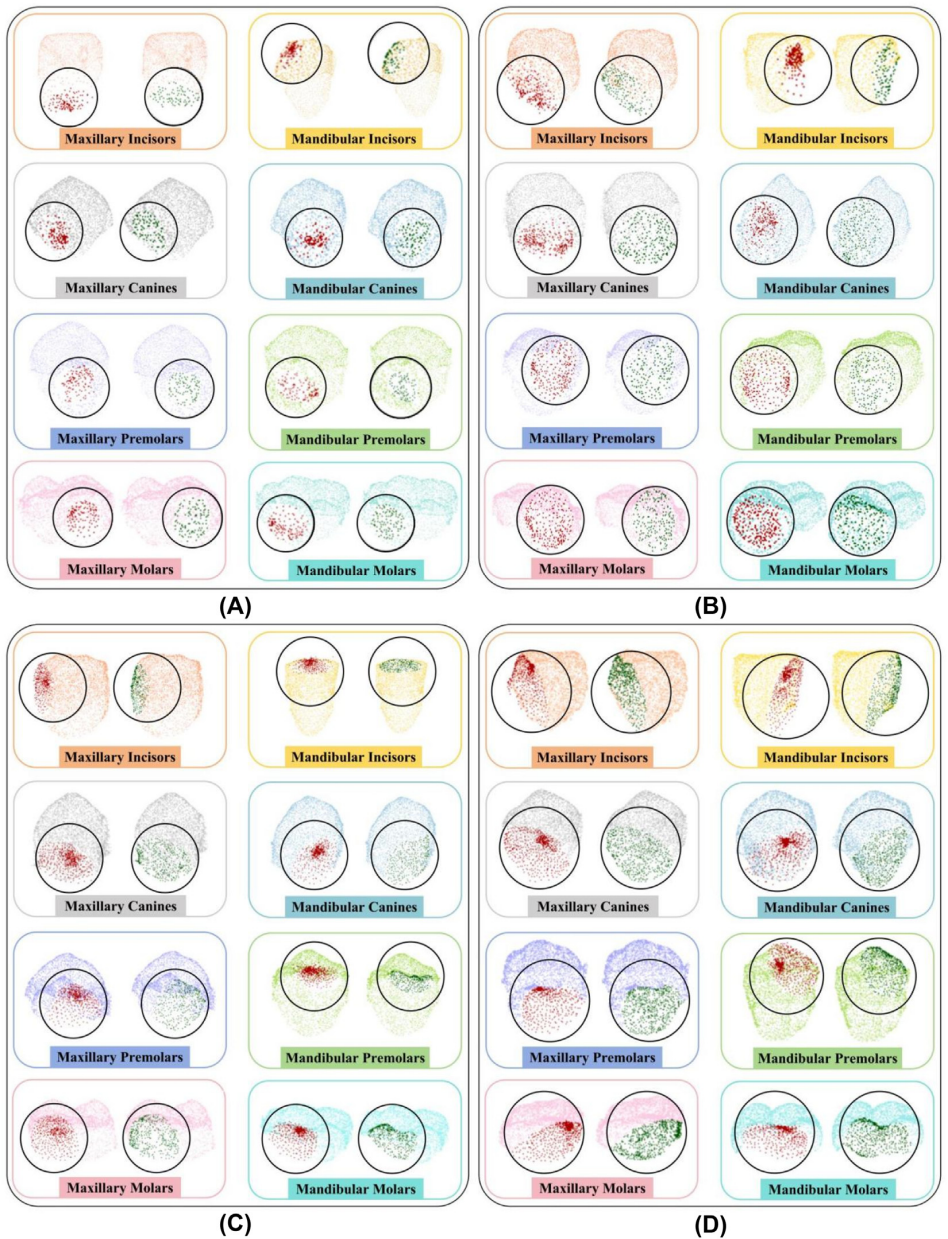
Visual comparison between the completion results generated by our proposed method and the ground truth under different missing proportions. Green points represent the ground truth of the missing regions, while red points represent the completion results generated by our method. (A) Missing 5%, (B) Missing 10%, (C) Missing 15%, and (D) Missing 20%.

**Fig. 6:**
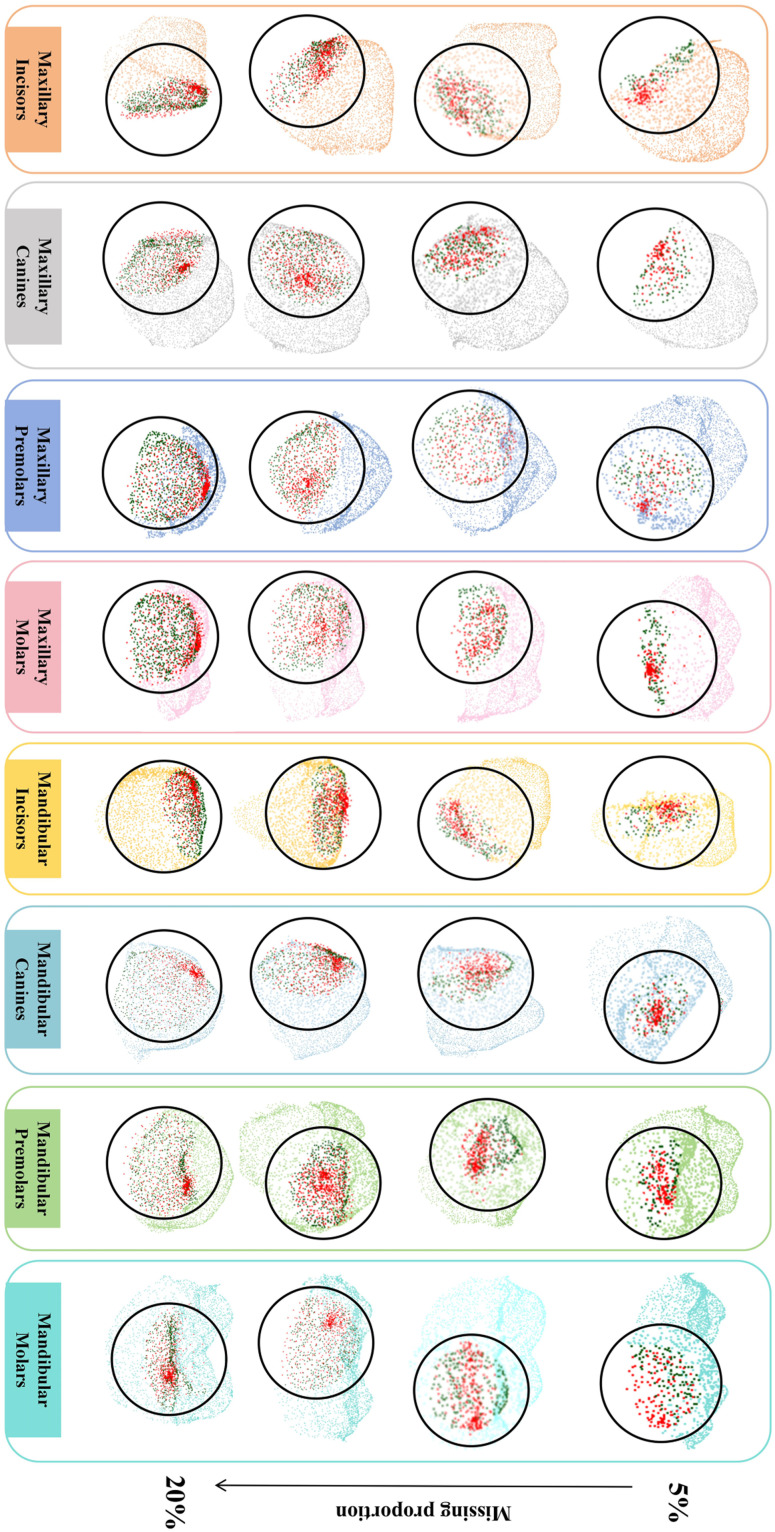
Visual overlay of the completion results generated by our proposed method and the ground truth under different missing proportions. Green points represent the ground truth of the missing regions, while red points represent the completion results generated by our method.

Furthermore, Figures 5 and 6 show a visual comparison of the completed results generated by the PF-Net–based model and the real point clouds for different tooth positions and missing proportions. As seen in the figure, while there may be some differences in the distribution of the point clouds between the generated and real point clouds, the overall position, size, and edge shape are closely aligned with the real values. This demonstrates the effectiveness of the model in completing the crown point clouds.

## Discussion

This study proposes a deep learning–based point cloud completion method, training and validating a deep learning model based on the PF-Net architecture to accurately and efficiently fill in missing regions of 3D point cloud data obtained from IOS. This architecture combines feature extraction through deep point cloud learning with the characteristics of Generative Adversarial Networks (GANs)^37^ to restore incomplete point cloud data. In the field of medical image processing, existing research typically applies GANs to the generation or reconstruction of 3D voxel data from computed tomography or magnetic resonance imaging scans.^38^ This model, however, is the first to apply GANs to the completion of 3D IOS data, demonstrating its feasibility and potential for application in the processing of 3D surface data.

During model training, when the missing rate is 5% or 10%, Loss_G and Loss_D exhibit some fluctuations in the later stages. This phenomenon may be attributed to the adversarial dynamics in GAN training.^39^ At these lower missing rates, rough or imperfect structures at the edges of the tooth crowns, such as the neck and adjacent surfaces, may mislead the generator during learning. As a result, the quality of the generated data fluctuates. Due to the instability in the generated data, the discriminator sometimes struggles to differentiate between real and fake data, while at other times, it can easily distinguish them, leading to fluctuations in its loss as well. In addition, when the number of generated points is relatively small, the impact of errors in generating or recognising edge noise points is more significant, affecting the overall loss of both the generator and discriminator, which contributes to the model's instability.

Similarly, the model testing results show that the highest CD value among the test samples appears at a 5% missing rate, while the lowest value is observed at a 10% missing rate. This may be because the model is less stable at the 5% missing rate, potentially leading to overfitting on the existing data features and thus reducing its ability to generalise effectively. In contrast, at a 20% missing rate, the generator faces greater challenges in reconstructing the missing data, which leads to decreased performance and an increased CD value. Therefore, the 15% missing rate represents an optimal balance in the adversarial training between the generator and discriminator, resulting in the best overall model performance. In addition, the HD results show a gradual increase as the missing data proportion grows. This indicates that the maximum point-wise deviation tends to rise with higher missing rates, although the changes remain relatively moderate. Overall, our proposed method excels at restoring missing regions in point clouds. It consistently achieves low and stable average deviations, while also maintaining good control over maximum deviations. The model effectively reconstructs missing areas in most test cases and still performs well even with 20% missing data. These results demonstrate the robustness and practical value of the method in handling varying levels of data loss, which is particularly important in clinical scenarios where IOS data may be unpredictably incomplete due to patient variability.

Current surveys indicate that a well-trained and experienced clinician or technician typically requires around 20 minutes to perform a full arch scan of both the upper and lower jaws for a single patient.^40^ This extended time is mainly due to the complex nature of the oral cavity, where many areas are often affected by local defects or distortions during the initial scan, requiring repeated scanning. The method proposed in this study requires approximately 0.5 seconds to restore a partially missing dental crown, thus meeting the real-time completion requirement. Therefore, if it is integrated into the IOS process, the scanner could simultaneously generate a complete 3D point cloud model during the scan. This ability would significantly reduce chairside time, enhancing the patient experience and decreasing the need for multiple scans. In addition, this efficiency would streamline the workflow for dental professionals, making the entire process faster and more precise. The automation of point cloud repair also reduces reliance on manual corrections or rescans, minimising human error and further increasing the reliability of the scanning process.

Despite its promising results, the current study has several limitations. Although the proposed method performs well in terms of accuracy, there are still subtle differences between the reconstructed point clouds and the ground truth, particularly in their distribution and edge morphology. Moreover, this study did not include a performance comparison between PF-Net and other state-of-the-art point cloud completion methods. Future work could focus on optimising the model and conducting comparative evaluations between PF-Net and other existing approaches, particularly in edge detection and surface continuity handling. Such efforts will allow for a more comprehensive assessment of the method and help enhance the overall quality of the reconstructed point clouds. In addition, while this study used a large dataset with various tooth types, the model's performance across different scanner brands or settings still requires further validation. As scanning technology continues to evolve, further testing of the model's adaptability to various scanner equipment will be essential. Furthermore, when dealing with more complex dental conditions, such as severe cavities or abnormal tooth shapes, the model may experience performance fluctuations. Future research should explore the model's adaptability to a broader range of clinical scenarios, ensuring its suitability for diverse patient groups and dental conditions.

Moreover, while this study focused on filling missing regions in dental point cloud data, the proposed method could be extended to other 3D medical imaging fields, such as facial scans or scans of other body parts, which also face data loss issues.^41^ The cross-domain potential of this technology opens up new possibilities for point cloud repair in various medical fields, especially as IOS technology continues to evolve and is increasingly used in clinical practice. By applying PF-Net to other medical imaging applications, particularly in addressing data loss and improving reconstruction accuracy, the technology could significantly enhance medical imaging workflows and clinical decision-making.

In summary, the proposed method demonstrated high accuracy and real-time point cloud completion, offering a promising advancement in IOS and data restoration. This technology has the potential to significantly enhance the efficiency and precision of dental care by automating point cloud repair and reducing reliance on manual interventions, making it a valuable tool for dental clinicians. Future research will further optimise the model and extend its application across a broader range of clinical scenarios, ensuring its robust performance and adaptability in diverse medical settings.

## Conclusion

This study introduces a deep learning–based method utilising the PF-Net model to effectively complete missing regions in 3D dental point clouds obtained from IOS. The model employs a multiresolution encoder–decoder framework that captures both low-level and high-level features of the partially missing dental point clouds, ensuring the preservation of the original geometric integrity during the completion process. The results demonstrate that our approach achieves high accuracy in restoring missing data of dental hard tissues, particularly for moderate levels of data loss, and is capable of generating complete point clouds in real time. This significantly reduces chairside time and enhances clinical efficiency.

## Author contributions

Qianhan Zheng: Conceptualisation, Methodology, Software, Validation, Formal analysis, Investigation, Writing - Original Draft, and Visualisation. Yimin Wang: Conceptualisation, Methodology, Software, Validation, Resources, Data Curation, and Writing - Original Draft. Mengqi Zhou: Conceptualisation, Methodology, Software, Validation, Resources, and Data Curation. Yongjia Wu: Conceptualisation, Software, Formal analysis, Investigation. Jiahao Chen: Methodology, Software, Resources, and Data Curation. Xiaozhe Wang: Validation, Formal analysis, and Investigation. Lixia Gao: Resources and Data Curation. Ting Kang: Conceptualisation, Writing - Review & Editing, Supervision. Weifang Zhang: Conceptualisation, Project administration, and Funding acquisition. Xuepeng Chen: Conceptualisation, Writing - Review & Editing, Visualisation, Supervision, Project administration, Funding acquisition.

## Funding

This work was supported by the Fundamental Research Funds for Central Universities (2023QZJH60); Key R&D Program of Zhejiang (2023C03072); the Funds of the Central Government Guiding Local Science and Technology Development (2023ZY1060); R&D Program of the Stomatology Hospital of Zhejiang University School of Medicine (RD2022DLYB03); Zhejiang Provincial Clinical Research Center (grant no. 2024-KFKT-03); and Zhejiang Province Medical and Health Science and Technology Plan (2024KY056). Xuepeng Chen is sponsored by the Zhejiang Provincial Program for the Cultivation of High-level Innovative Health Talents.

## Conflict of interest

The authors declare that they have no known competing financial interests or personal relationships that could have appeared to influence the work reported in this paper.

## References

[1] Shah N., Thakur M., Gill S. (2023). Validation of digital impressions' accuracy obtained using intraoral and extraoral scanners: a systematic review. J Clin Med.

[2] Abduo J., Elseyoufi M. (2018). Accuracy of intraoral scanners: a systematic review of influencing factors. Eur J Prosthodont Restor Dent.

[3] Ma J., Zhang B., Song H., Wu D., Song T. (2023). Accuracy of digital implant impressions obtained using intraoral scanners: a systematic review and meta-analysis of in vivo studies. Int J Implant Dent.

[4] Mizumoto R.M., Yilmaz B. (2018). Intraoral scan bodies in implant dentistry: a systematic review. J Prosthet Dent.

[5] Joda T., Brägger U. (2016). Patient-centered outcomes comparing digital and conventional implant impression procedures: a randomized crossover trial. Clin Oral Implants Res.

[6] Mangano F.G., Hauschild U., Veronesi G., Imburgia M., Mangano C., Admakin O. (2019). Trueness and precision of 5 intraoral scanners in the impressions of single and multiple implants: a comparative in vitro study. BMC Oral Health.

[7] Chiu A., Chen Y.W., Hayashi J., Sadr A. (2020). Accuracy of CAD/CAM digital impressions with different intraoral scanner parameters. Sensors (Basel).

[8] Tallarico M., Xhanari E., Kim Y.J. (2019). Accuracy of computer-assisted template-based implant placement using conventional impression and scan model or intraoral digital impression: a randomised controlled trial with 1 year of follow-up. Int J Oral Implantol (Berl).

[9] Liu W., Sun J., Li W., Hu T., Wang P. (2019). Deep learning on point clouds and its application: a survey. Sensors (Basel).

[10] Guo Y., Wang H., Hu Q., Liu H., Liu L., Bennamoun M. (2021). Deep learning for 3D point clouds: a survey. IEEE Trans Pattern Anal Mach Intell.

[11] Flügge T.V., Schlager S., Nelson K., Nahles S., Metzger MC. (2013). Precision of intraoral digital dental impressions with iTero and extraoral digitization with the iTero and a model scanner. Am J Orthod Dentofacial Orthop.

[12] Wesemann C., Kienbaum H., Thun M., Spies B.C., Beuer F., Bumann A. (2021). Does ambient light affect the accuracy and scanning time of intraoral scans?. J Prosthet Dent.

[13] Papaspyridakos P., Chen Y.W., Alshawaf B. (2020). Digital workflow: in vitro accuracy of 3D printed casts generated from complete-arch digital implant scans. J Prosthet Dent.

[14] Samaranayake L., Tuygunov N., Schwendicke F., Osathanon T., Khurshid Z., Boymuradov S.A., Cahyanto A. (2025). The transformative role of artificial intelligence in dentistry: a comprehensive overview. Part 1: fundamentals of AI, and its contemporary applications in dentistry. Int Dent J.

[15] Tuygunov N., Samaranayake L., Khurshid Z. (2025). The transformative role of artificial intelligence in dentistry: a comprehensive overview. Part 2: the promise and perils, and the International Dental Federation Communique. Int Dent J.

[16] Su H., Maji S., Kalogerakis E., Erik L. (2015). Proceedings of the IEEE International Conference on Computer Vision.

[17] Drokin I., Ericheva E. (2020). International Conference on Analysis of Images, Social Networks and Texts.

[18] Qi C.R., Su H., Mo K., Guibas LJ. (2017). Proceedings of the IEEE Conference on Computer Vision and Pattern Recognition.

[19] Qi C.R., Yi L., Su H., Guibas LJ. (2017). Pointnet++: deep hierarchical feature learning on point sets in a metric space. Adv Neural Inf Process Syst.

[20] Li Y., Bu R., Sun M., Wu W., Di X., Chen B. (2018). Pointcnn: convolution on x-transformed points. Adv Neural Inf Process Syst.

[21] Wang Y., Sun Y., Liu Z., Sarma S.E., Bronstein M.M., Solomon JM. (2019). Dynamic graph CNN for learning on point clouds. ACM Trans Graph.

[22] Guo M.H., Cai J.X., Liu Z.N., Mu T.J., Martin R.R., Hu SM. (2021). Pct: point cloud transformer. Comp Visual Media.

[23] Zanjani F.G., Moin D.A., Verheij B. (2019). Proceedings of the 2nd International Conference on Medical Imaging with Deep Learning.

[24] Guo C., Dai N., Tian S.K. (2020). Morphological design of missing tooth driven by high-resolution deep generation network. J Image Graph.

[25] Huang Z., Yu Y., Xu J., Ni F., Le X. (2020). Proceedings of the 2020 IEEE/CVF Conference on Computer Vision and Pattern Recognition.

[26] Lian C., Wang L., Wu T.H. (2020). Deep multi-scale mesh feature learning for automated labeling of raw dental surfaces from 3D intraoral scanners. IEEE Trans Med Imaging.

[27] Hao R., Wei Z., He X. (2022). Multistage adaptive point-growth network for dense point cloud completion. Remote Sens.

[28] Li W., Pan J., Hasegawa K., Li L., Tanaka S. (2024). Missing region completion network for large-scale laser-scanned point clouds: application to transparent visualization of cultural heritage. Remote Sens.

[29] Xu J., Zhang Y., Zou Y., Liu P.X. (2024). Sequence generation completion method and resolution scaling network for point cloud completion. IEEE Trans Geosci Remote Sens.

[30] Achlioptas P., Diamanti O., Mitliagkas I., Guibas L.J. (2018). International Conference on Machine Learning.

[31] Lin T.Y., Dollar P., Girshick R. (2017). Proceedings of the IEEE Conference on Computer Vision and Pattern Recognition.

[32] Yang Y., Feng C., Shen Y., Tian D. (2018). Proceedings of the IEEE Conference on Computer Vision and Pattern Recognition.

[33] Fan H., Hao S., Guibas L. (2017). Proceedings of the IEEE Conference on Computer Vision and Pattern Recognition.

[34] Lin C., Kong C., Lucey S. (2018). Proceedings of the AAAI Conference on Artificial Intelligence.

[35] Gadelha M., Wang R., Maji S. (2018). Proceedings of the European Conference on Computer Vision.

[36] Aydin O.U., Taha A.A., Hilbert A. (2021). On the usage of average Hausdorff distance for segmentation performance assessment: hidden error when used for ranking. Eur Radiol Exp.

[37] Goodfellow I., Pougetabadie J., Mirza M. (2014). Generative adversarial nets. Adv Neural Inf Process Syst.

[38] Zhang F., Wang L., Zhao J., Zhang X. (2023). Medical applications of generative adversarial network: a visualization analysis. Acta Radiol.

[39] Li C., Xu K., Zhu J., Liu J., Zhang B. (2022). Triple generative adversarial networks. IEEE Trans Pattern Anal Mach Intell.

[40] Siqueira R., Galli M., Chen Z. (2021). Intraoral scanning reduces procedure time and improves patient comfort in fixed prosthodontics and implant dentistry: a systematic review. Clin Oral Investig.

[41] Park J.H., Lee G.H., Moon D.N., Yun K.D., Kim J.C., Lee KC. (2022). Creation of digital virtual patient by integrating CBCT, intraoral scan, 3D facial scan: an approach to methodology for integration accuracy. J Craniofac Surg.

